# Sweet Breath

**Published:** 1873-02

**Authors:** 


### SWEET BREATH.

Foul breath may be occasioned by a decaying tooth, by dyspepsia, or it may arise from a scrofulous constitution; in each case a radical cure can be promised only on the removal of the cause. Yet there are times when it may be specially desirable to be free from any odor of an offensive kind; in such cases have on hand as a part of the toilet a two-ounce vial of the concentrated solution of the "chloride of soda." To two table-spoonfuls of water add ten drops of the soda, and rinse the mouth out freely before leaving the house. Take a mouthful and keep it in for two or three minutes, and repeat.

If there are ill-smelling feet or odor from under the arm take some of the same preparation in the hollow of the hand, and rub it well into the skin of the parts; if none at hand, use spirits of camphor or camphor-water. But persons who have ill-smelling feet should wash them well every night with soap and warm water.

